# Hereditary Thrombotic Thrombocytopenic Purpura in a Chinese Boy With a Novel Compound Heterozygous Mutation of the *ADAMTS13* Gene

**DOI:** 10.3389/fped.2020.00554

**Published:** 2020-09-10

**Authors:** Yi-ling Dai, Xue Tang, Hong-bo Chen, Qiu-yu Peng, Xia Guo, Ju Gao

**Affiliations:** ^1^Department of Pediatrics, West China Second University Hospital, Sichuan University, Sichuan, China; ^2^Key Laboratory of Birth Defects and Related Diseases of Women and Children, Sichuan University, Ministry of Education, Sichuan, China

**Keywords:** hereditary thrombotic thrombocytopenic purpura, Upshaw-Schulman syndrome, *ADAMTS13*, novel mutation, Chinese

## Abstract

Hereditary thrombotic thrombocytopenic purpura (TTP) is caused by *ADAMTS13* mutations with autosomal recessive inheritance. It typically presents during childhood and is frequently misdiagnosed as immune thrombocytopenia. We present a case of hereditary TTP with an undescribed compound heterozygous *ADAMTS13* mutation in a Chinese boy. A 12-year-old boy with a history of intermittent thrombocytopenia in the prior 6 years had severe deficiency of plasma ADAMTS13 and harbored a novel compound heterozygous mutation which was also identified in his sister. The c.577C>T was a pathogenic variant reported exclusively in Japanese cases. The undescribed c.2397C>A non-sense mutation was predicted to encode a truncated protein. Identification of the specific novel heterozygous *ADAMTS13* mutation in the Chinese family, consisting a variant restricted to Asian individuals and an undescribed c.2397C>A non-sense mutation, demonstrates genetic diversity underlying hereditary TTP, and possibly ethnic skewed mutation profiles.

## Background

Hereditary thrombotic thrombocytopenic purpura (TTP), also known as Upshaw–Schulman syndrome (USS), is the congenital form of TTP, with an annual incidence of less than 1 per million (1), caused by mutations of the *ADAMTS13* (A Disintegrin And Metalloproteinase with a ThromboSpondin type 1 motif, member 13) gene encoding von Willebrand factor (vWF)-cleaving protease (2, 3). *ADAMTS13* mutations with resultant reduced synthesis or increased clearance lead to severe deficiency of ADAMTS13, failure of cleavage of ultralarge VWF multimers, platelet hyperaggregation, and consumptive thrombocytopenia (4, 5). USS and acquired immune-mediated TTP are both characterized by Coombs test-negative microangiopathic hemolytic anemia, consumptive thrombocytopenia, and a variety of organ damages, and both are categorized under the heading of thrombotic microangiopathy (TMA) (6, 7). Over 200 mutations associated with USS have been reported, which differ in different geographical areas and ethnic groups (1). Here, we present a novel compound heterozygous mutation which has not been described, in a Chinese boy and his older sister.

## Case Report

The patient, a 12-year-old boy, firstly presented with petechiae over his trunk and extremities for no obvious reasons at age of six. Isolated thrombocytopenia was revealed with platelet counts ranging from 20 to 30 × 10^9^/L in local hospitals. A diagnosis of primary immune thrombocytopenia (ITP) was made, and oral steroid therapy was prescribed for several months. He had remained symptom-free for 4 years, and his platelet counts were within the normal ranges thereafter. Then, he suffered several acute attacks of severe thrombocytopenia (lowest platelet count of 5.0 × 10^9^/L) with concomitant anemia (lowest Hb concentration being 62 g/L), without renal symptoms, neurological symptoms, headaches, abdominal pain, and lethargy, typically triggered by upper respiratory infections. Bone marrow aspiration showed markedly increased megakyrocytes and maturation arrest. Diagnoses of primary ITP and bleeding anemia were made and steroid, intravenous immunoglobulin, blood, and platelet transfusions were given when indicated in other hospitals, but platelet counts were still below normal. The patient presented to our hospital on 31 December 2018, because of thrombocytopenia (platelet count 1.0 × 10^9^/L) and epitaxis. Careful history-taking and relevant laboratory investigations strongly suggested the possibility of thrombotic microangiopathy, because the patient suffered thrombocytopenia, anemia which could not be explained by hemorrhage, conspicuous reticulocytosis (15.2%), and remarkably elevated LDH levels (2,357 IU/L) during acute attacks. Coombs test, DIC screening tests, and liver and renal functions were negative; urinary trace proteins were positive. His old sister suffered thrombocytopenia at 5 years of age but remained disease-free thereafter. Parental consanguinity was denied. Massive plasma infusions (3,600 mL during the 4-day therapy) were given on an emergency basis, which successfully stopped epitaxis, and platelet count was normalized on the third day. Plasma ADAMTS13 enzymatic activity assayed by the SELDI-TOF (surface-enhanced laser resorption/ionization-time of flight) method in a third-party laboratory after the plasma infusion (600 mL) was 32.1%, while the platelet count was 86 × 10^9^/L. A repeated assay 2 weeks after the last plasma infusion returned an enzymatic activity of <5%, and the platelet count was 43 × 10^9^/L. Assay of ADAMTS13 autoantibody was not available at the time of initial diagnosis of USS.

**Figure 1:**
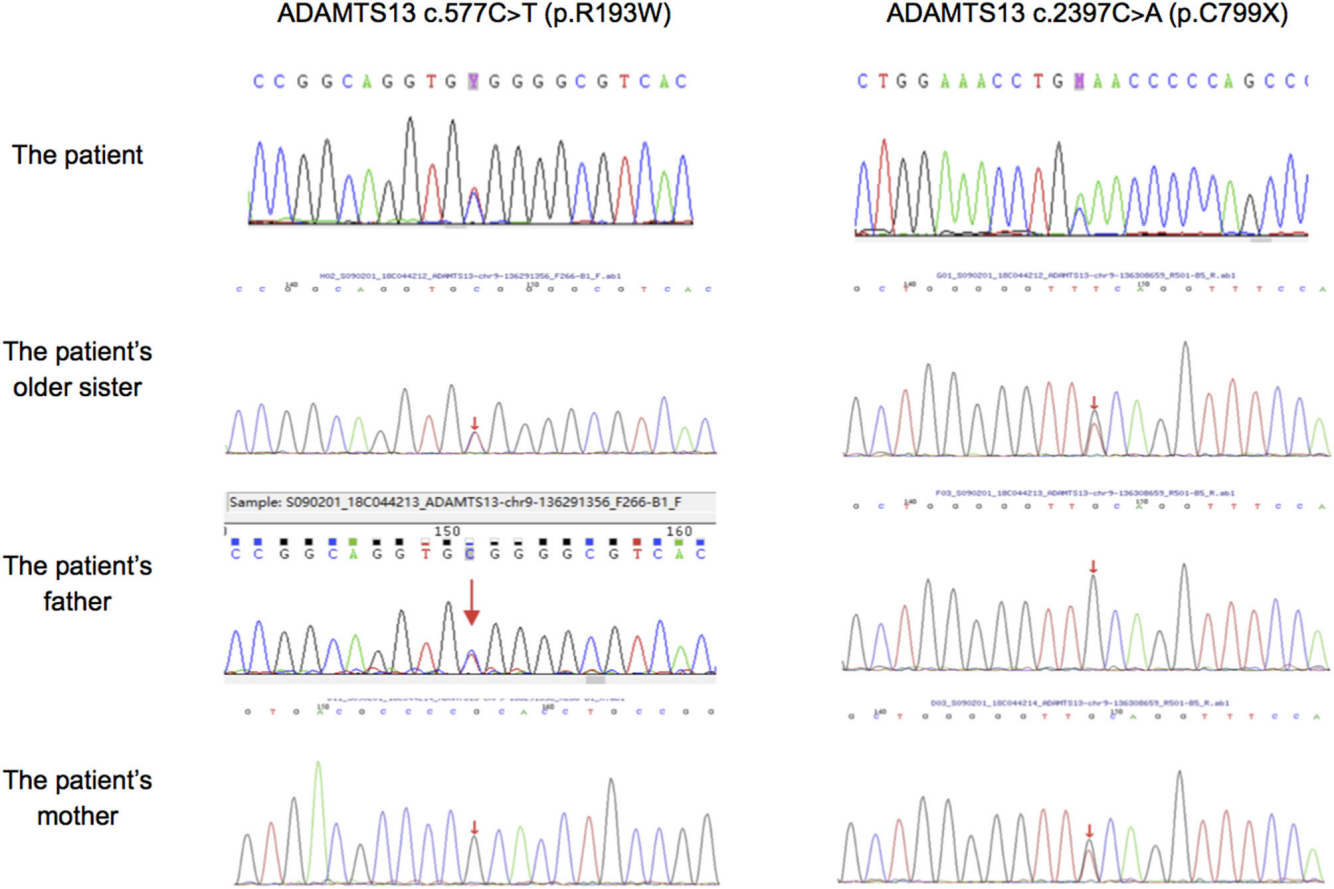
Sanger sequencing results in *ADAMTS13* of our patient, his older sister and his parents. The patient, his older sister and his farther had mutation of c.577C>T, while the patient, his older sister and his mother had mutation of c.2397C>A.

**Figure 2:**
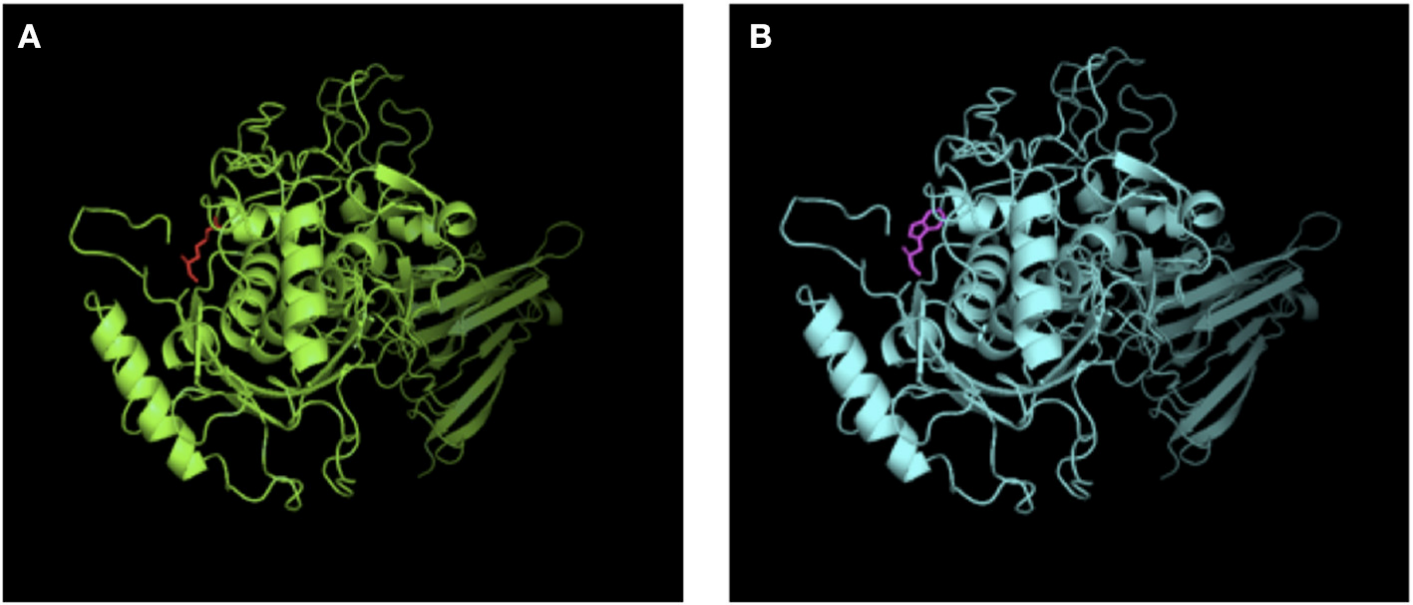
SWISS-MODEL-predicted structures of ADAMST13^WT^ **(A)** and ADAMST13 ^p.R193W^ **(B)**. Tryptophan (a basic amino acid with molecular weight of 204) in substitution for arginine (an aromatic amino acid, with molecular weight of 174) changed the molecular weight, polarization and folding, probably leading to accelerated protein degradation.

Mutation analysis revealed that both the patient and his sister harbored the same compound heterozygous *ADAMTS13* mutation: c.2397C>A and c.577C>T, inherited from his mother and father, respectively (Figure 1). The pathogenic c.577C>T (p.R193W) variant within exon 6 had been previously reported (8, 9). Molecular modeling analysis using the SWISS-MODEL indicated that tryptophan (a basic amino acid with molecular weight of 204) in substitution for arginine (an aromatic amino acid, with molecular weight of 174) changed the molecular weight, polarization, and folding, probably leading to accelerated protein degradation (Figure 2). While the novel c.2397C>A (p.C799X), a previously undescribed non-sense mutation, created a stop codon and encoded a truncated ADAMTS13 protein. Therefore, a definitive diagnosis of USS caused by a novel compound heterozygous mutation in this Chinese boy was firmly established.

## Discussion

Hereditary thrombotic thrombocytopenic purpura (TTP), or Upshaw–Schulman syndrome (USS), is an exceedingly rare hereditary disorder, which presents primarily in childhood, constituting up to 5% of TTP (1, 9). Genetically, USS is caused by *ADAMTS13* gene mutations in an autosomal recessive mode of inheritance and is more prevalent in families of consanguinity. Biochemically, USS is characterized by severe constitutional deficiency of plasma ADAMTS13, generally less than 10% of normal, which results in accumulation of hyperadhesive ultra-large vWF (ULVWF) multimers and triggers activation of the VWF-platelet hemostatic pathway, culminating in platelet-ULVWF microthrombi formation within microcirculation (3, 6), and currently categorized as *ADAMTS13* gene mutation-associated vascular microthrombotic disease (VMTD) (10, 11).

More than 200 *ADAMTS13* mutations spreading in all protein domains have been identified in patients with USS, with splicing mutations being increasingly reported (1). Nevertheless, type and frequency of genotypes are highly diverse, with some specific gene variants particularly common in certain ethnic populations. Mutation analysis of a German cohort with 30 USS patients identified c.4143_4144dupA (p.Glu1382Arg fs^*^6) homozygotes in 4 patients and heterozygotes in another 10 patients (12), suggesting a common mutation origin in individuals of northern European ancestry (13). Similarly, the allelic variant c.577C>T (p.R193W) in our index patient was exclusively identified in Japanese patients enrolled in the International Hereditary Thrombotic Thrombocytopenic Purpura Registry and the most prevalent pathogenic mutation in Asia (14), illustrating genetic-background skewed mutation profiling.

USS has been rarely reported in China, which is not a participating country of the International USS registry. With extensive literature search, we just identified a couple of case reports and a small 5-case study in children with genetically confirmed USS, the largest case report in China (15). Nevertheless, the novel mutation of our index patient has not been described in USS patients.

ADAMTS13 deficiency could result from reduced synthesis, impaired proteolytic activity, or increased degradation. *In vitro* functional analysis of recombinant *ADAMTS13* mutants indicates that mutations involving the metalloprotease catalytic domain generally results in severely decreased enzyme activity, with or without impaired secretion (12). Pathogenic c.577C>T missense mutation in exon 6 inactivates metalloprotease, probably adversely affects protein misfolding, and accelerates proteins degradation (Figure 2) (16). The previously undescribed c.2397C>A (p.C799X) non-sense mutation in exon 19 involving the TSP1-3 domain encodes a truncated ADAMTS13 protein likely degraded rapidly by NMD (non-sense-mediated RNA decay) (12) and judged as pathogenic according to the ACMG guideline.

Clinically, USS is highly heterogeneous in terms disease onset, manifestations, and prognosis. Most patients present in childhood, but some have their first episodes in adulthood, particularly during pregnancies (17), which are somewhat correlated with specific mutation profiles (18). In addition, disease manifestations and severity differ remarkably and there are no definitive correlations between genotypes and phenotypes. Six patients out of 30 in the German cohort had a mild clinical course defined as recurrent episodes of isolated thrombocytopenia without any organ involvement in spite of severe ADAMTS13 deficiency of less 6% in mild cases (12). In addition, patients with the same gene mutations could have quite different clinical pictures (12), including patients from the same family (19), as illustrated by our index case and his old sister who just experienced one single attack of isolated thrombocytopenia, suggesting other disease-modifying environmental or genetic factors.

USS can remain symptom-free for years between episodes and is most frequently triggered by infections and other inciting events (1, 14). Therefore, USS are easily misdiagnosed as primary immune thrombocytopenia, especially during childhood. In fact, 12 patients were initially misdiagnosed before 7 years as idiopathic immune thrombocytopenia, and 13 cases as TTP or other disease entities, out of an early Japanese cohort with 43 USS patients (20). The International USS Registry study clearly identified a significant delay of USS diagnosis, with median ages of overt presentation and clinical diagnosis being 4.5 and 16.7 years, respectively (14).

The differential diagnosis of USS from immune thrombocytopenia is of great clinical implication. The correct diagnosis of USS should be based on relevant family history, clinical presentations, plasma ADAMTS13 determination, and possibly mutation analysis. This helps to promptly institute plasma therapy to control acute attacks and prevent organ damages, to plan genetic counseling for family members, and to avoid unnecessary immunosuppressive therapy (1, 5, 21).

Prophylactic plasma given at 1- to 2-week intervals is a substantial measure to prevent recurrent episodes. However, prophylactic plasma infusion requires clinic visits and establishment of venous access. In addition, recombinant ADAMTS13 has been documented to be safe and could be self-administered at home (22).

In summary, we present an undescribed compound heterozygous *ADMATS13* mutation, adding new information to the USS mutation database. Further studies are needed to explore ethnic and functional aspects of this specific variant.

## Data Availability Statement

The original contributions presented in the study are included in the article/Supplementary Material, further inquiries can be directed to the corresponding author/s.

## Ethics Statement

Ethical review and approval was not required for the study on human participants in accordance with the local legislation and institutional requirements. Written informed consent to participate in this study was provided by the participants' legal guardian/next of kin.

## Author Contributions

YD and XT: writing—original draft. JG: writing—review & editing. HC: processing pictures. QP: data curation. XG and JG: project administration. All authors have read and approved the final manuscript.

## Conflict of Interest

The authors declare that the research was conducted in the absence of any commercial or financial relationships that could be construed as a potential conflict of interest.
